# Supplementary material for: Content-rich biological network constructed by mining PubMed abstracts
Source: BMC Bioinformatics. 2004 Oct 8;5:147. doi: 10.1186/1471-2105-5-147 (PMC528731; doi:10.1186/1471-2105-5-147)
Supplement: Additional File 2 — The original results of the above study (non-essential files are deleted to keep the file size under the limit set by BMC bioinformatics). [file 1471-2105-5-147-S2.bz2 › chilibotAdditionalFile2/dip50/376ID10713102E9789/html/NGFB_NTRK1.html]

 


 **NGFB** and **NTRK1** 
  
Found 1439 abstracts in PubMed, retrieved 50.  
 

 What does Google say? 
 PDF only 
| .edu only 

---

**Interactive relationship** (e.g. stimulation, inhibition, etc)

**Inhibitory relationship**- Methylmercury Hg at 30 nM for neurite outgrowth inhibition did not affect NGF  [ **NGFB** ]  binding to TrkA  [ **NTRK1** ] .  Ref: 12644250 Brain Res Dev Brain Res, 2003
- Concurrent exposure to CHHg and NGF  [ **NGFB** ]  for 2.5 min resulted in a concentration dependent decrease in TrkA  [ **NTRK1** ]  autophosphorylation, which was significant at 100 nM CHHg.  Ref: 12644250 Brain Res Dev Brain Res, 2003
- RESULTS These results revealed a low amount of NGF  [ **NGFB** ]  in the plasma of old subjects associated with a reduced expression of mRNA for NGF  [ **NGFB** ]  receptor trkA  [ **NTRK1** ]  in peripheral lymphocytes.  Ref: 12679610 Gerontology< MedlineTA>Gerontology, 2003
- Pathways responsible for transducing the sensitisation of TRPV1 by TrkA  [ **NTRK1** ] , the NGF  [ **NGFB** ]  receptor, were characterised by observing the effects of inhibitors of key members of NGF  [ **NGFB** ]  activated second messenger signalling cascades.  Ref: 12815188 J Physiol< MedlineTA>J Physiol, 2003
- A gradient of TrkA  [ **NTRK1** ]  and NGF  [ **NGFB** ]  expression exists and is highest in cord blood CD34 cells, reduced in cord blood mononuclear cells MNC and minimal in mononuclear cells isolated from adult peripheral blood.Our findings suggest that NGF  [ **NGFB** ]  may play a role in the differentiation of hematopoietic progenitors and indicate a different requirement for NGF  [ **NGFB** ]  by immune cells, depending on their state of maturity.  Ref: 12620652 J Neuroimmunol, 2003
- The p75 NTR transfection only resulted in the mild differentiation response, and transfection of TrkA  [ **NTRK1** ]  gene caused remarkable neurite extension, up regulation of neurofilament and decreased expression of N myc oncogene after NGF  [ **NGFB** ]  treatment.  Ref: 12877805 Chin Med J (Engl), 2003
**Stimulatory relationship**- Our results suggest that CHHg may inhibit differentiation of PC12 cells by interfering with NGF  [ **NGFB** ]  stimulated TrkA  [ **NTRK1** ]  autophosphorylation.  Ref: 12644250 Brain Res Dev Brain Res, 2003
- The TRK  [ **NTRK1** ]  oncoproteins signal transduction has been in part elucidated, and it involves several signal transducers activated by the NGF  [ **NGFB** ]  stimulated  **NTRK1**  receptor.  Ref: 12586769 Endocrinology, 2003
- To address this issue, various combinations of transfected PC12 cells expressing a PDGFR TrkA  [ **NTRK1** ]  chimera and the p75NTR selective NGF  [ **NGFB** ]  mutant D9 13 NGF  [ **NGFB** ]  were utilized to selectively stimulate TrkA  [ **NTRK1** ]  or p75NTR signaling, respectively.  Ref: 12702729 J Biol Chem< MedlineTA>J Biol Chem, 2003
- It is similar to the one reported to relate with p75 and TrkA  [ **NTRK1** ]  receptors and stimulated by the prototypic NT, nerve growth factor  [ **NGFB** ] .  Ref: 12616526 J Cell Biochem, 2003
**Neutral relationship**- Degrees of down regulation in NGF  [ **NGFB** ]  family were greater than those in their receptors Trk  [ **NTRK1** ]  family.  Ref: 12854135 World J Gastroenterol, 2003
- Protective effect of HSV mediated gene transfer of nerve growth factor  [ **NGFB** ]  in pyridoxine neuropathy demonstrates functional activity of trkA  [ **NTRK1** ]  receptors in large sensory neurons of adult animals.  Ref: 12603263 Eur J Neurosci, 2003
- The biological effects of Nerve Growth Factor  [ **NGFB** ]  NGF  [ **NGFB** ]  are primarily mediated via its high affinity receptor TrkA  [ **NTRK1** ] .  Ref: 12825322 Arch Ital Biol, 2003
- NGF  [ **NGFB** ]  modifies cholinergic neurons through its low p75 and high affinity TrkA  [ **NTRK1** ]  receptors.  Ref: 12675131 Neurochem Res, 2003
- On the other hand, the up regulated astroglial TrkA  [ **NTRK1** ]  receptor may be an important target for exogenous NGF  [ **NGFB** ] , which, as previously described J Neurosci 11 1991 2914.  Ref: 12849738 Neuroscience, 2003
- CONCLUSIONS Coexpression of NGF  [ **NGFB** ]  with molecules involved in angiogenesis and p TrkA  [ **NTRK1** ]  expression in endothelial cells suggest that the proangiogenic role attributed to NGF  [ **NGFB** ]  in vitro and in vivo may be relevant in clinical cancer.  Ref: 12796393 Clin Cancer Res< MedlineTA>Clin Cancer Res, 2003
- Here we show that survival of sympathetic neurons supported by NGF  [ **NGFB** ]  on distal axons requires the kinase activity of the NGF  [ **NGFB** ]  receptor, TrkA  [ **NTRK1** ] , in both distal axons and cell bodies.  Ref: 12848932 Neuron, 2003
- The nerve growth factor  [ **NGFB** ]  NGF  [ **NGFB** ]  family of neurotrophins binds two classes of cell surface receptors, trk  [ **NTRK1** ]  receptor tyrosine kinases and the shared p75 receptor.  Ref: 12716928 J Neurosci, 2003
- Surprisingly, NGF  [ **NGFB** ]  initiated intracellular targeting of DOR in PC12 cells acutely, within 60 min after initial activation of TrkA  [ **NTRK1** ] .  Ref: 12657666 J Neurosci, 2003
- Trk  [ **NTRK1** ]  receptors are a family of three receptor tyrosine kinases, each of which can be activated by one or more of four neurotrophins nerve growth factor  [ **NGFB** ] , brain derived neurotrophic factor, and neurotrophins 3 and 4.  Ref: 12676795 Annu Rev Biochem< MedlineTA>Annu Rev Biochem, 2003
- MTSV1 7 trk  [ **NTRK1** ]  neu and Hb2 trk  [ **NTRK1** ]  neu cells were transiently transfected with a construct in which the MUC1 promoter caused expression of a CAT reporter gene, and were then treated with NGF  [ **NGFB** ] .  Ref: 12798775 Cell Biol Int, 2003
- The distinct distribution of trkA  [ **NTRK1** ]  receptors on small neurons and trkC receptors on large neurons in the dorsal root ganglion correlates with the dependence of these two classes of neurons on nerve growth factor  [ **NGFB** ]  and neurotrophin 3, respectively, for survival during development.  Ref: 12603263 Eur J Neurosci, 2003

**Non-interactive relationship** (e.g. studied together, co-existance, homology, etc.)

- NGF  [ **NGFB** ] , BDGF, NT 3, NT 4 5, NT 6 and TrkA  [ **NTRK1** ] , B and C were down regulated simultaneously in gastric carcinoma in comparison with normal gastric mucosa.  Ref: 12854135 World J Gastroenterol, 2003
- Both flat and process bearing ECs were immunopositive for brain derived neurotrophic factor, nerve growth factor  [ **NGFB** ] , neurotrophin 4, and TrkB but not TrkA  [ **NTRK1** ] .  Ref: 12555206 Glia, 2003
- Here, we provide in vivo and in vitro evidence for the presence within DRG neurons of endosomes containing NGF  [ **NGFB** ] , activated TrkA  [ **NTRK1** ] , and signaling proteins of the Rap1 Erk1 2, p38MAPK, and PI3K Akt pathways.  Ref: 12848933 Neuron, 2003
- A possible role for NGF  [ **NGFB** ]  was suggested by the findings that parotid cells express both TrkA  [ **NTRK1** ]  and p75 receptors.  Ref: 12732363 Biochem Pharmacol, 2003
- SHB mediates certain responses in platelet derived growth factor PDGF receptor, fibroblast growth factor FGF receptor, neural growth factor NGF  [ **NGFB** ]  receptor TRKA  [ **NTRK1** ] , T cell receptor, interleukin 2 IL 2 receptor and focal adhesion kinase FAK signaling.  Ref: 12776987 Curr Mol Med, 2003
- Expression and Activation of the Nerve Growth Factor  [ **NGFB** ]  Receptor TrkA  [ **NTRK1** ]  in Serous Ovarian Carcinoma.  Ref: 12796393 Clin Cancer Res< MedlineTA>Clin Cancer Res, 2003
- Finally, these calculations, used along with the mutagenesis data, provide a basis for designing new peptides that mimic NGF  [ **NGFB** ]  in TrkA  [ **NTRK1** ]  binding function.  Ref: 12668437 Biophys J, 2003
- Experimental Design Sections from 80 malignant effusions and 65 corresponding solid tumors were evaluated for protein expression of NGF  [ **NGFB** ] , TrkA  [ **NTRK1** ] , and phospho TrkA  [ **NTRK1** ]  p TrkA  [ **NTRK1** ] .  Ref: 12796393 Clin Cancer Res< MedlineTA>Clin Cancer Res, 2003
- In addition, we wished to study the possible link between expression of NGF  [ **NGFB** ] , a novel angiogenic factor and its receptor TrkA  [ **NTRK1** ] , and the expression of factors involved in angiogenesis in effusions and solid tumors.  Ref: 12796393 Clin Cancer Res< MedlineTA>Clin Cancer Res, 2003
- METHODS Retroviral vectors were constructed to express the high affinity NGF  [ **NGFB** ]  receptor TrkA  [ **NTRK1** ]  and low affinity NGF  [ **NGFB** ]  receptor p75 NTR.  Ref: 12877805 Chin Med J (Engl), 2003
- To determine whether the observed inhibition of TrkA  [ **NTRK1** ]  was sufficient to alter cell differentiation, NGF  [ **NGFB** ]  stimulated neurite outgrowth was examined in PC12 cells after exposure to 30 nM CHHg, a concentration that inhibited TrkA  [ **NTRK1** ]  autophosphorylation by approximately 50%.  Ref: 12644250 Brain Res Dev Brain Res, 2003
- We propose that mitochondrial transport and or docking are regulated in part via NGF  [ **NGFB** ]  TrkA  [ **NTRK1** ]  PI 3 kinase signaling.  Ref: 12756280 J Exp Biol, 2003
- Brn3a regulation of TrkA  [ **NTRK1** ]  NGF  [ **NGFB** ]  receptor expression in developing sensory neurons.  Ref: 12810599 Development< MedlineTA>Development, 2003
- RESULTS NGF  [ **NGFB** ] , TrkA  [ **NTRK1** ] , and p TrkA  [ **NTRK1** ]  were expressed in carcinoma cells in effusions in 60 of 80 75%, 64 of 80 80%, and 15 of 80 19% specimens, respectively.  Ref: 12796393 Clin Cancer Res< MedlineTA>Clin Cancer Res, 2003
- However, NGF  [ **NGFB** ]  rescue from growth factor withdrawal requires NGF  [ **NGFB** ]  signalling through the high affinity tyrosine kinase receptor  [ **NTRK1** ]  TrkA  [ **NTRK1** ] , while NGF  [ **NGFB** ]  rescue from TNFalpha promoted cell death requires NGF  [ **NGFB** ]  signalling through the low affinity p75NTR receptor.  Ref: 12882321 Aging Cell, 2003
- NGF  [ **NGFB** ]  family and their receptors Trk  [ **NTRK1** ]  family probably play a unique role in gastric cancer cell apoptosis by a novel Ras or Raf signal transduction pathway.  Ref: 12854135 World J Gastroenterol, 2003
- To determine which of the neurotrophins NTs nerve growth factor  [ **NGFB** ]  NGF  [ **NGFB** ] , brain derived neurotrophic factor BDNF, neurotrophin 3 NT3, and neurotrophin 4 5 NT4 and their receptors NTrs, TrkA  [ **NTRK1** ] , TrkB, TrkC, and p75, are present in the adult rat lacrimal gland.  Ref: 12882781 Invest Ophthalmol Vis Sci, 2003
- CD34 positive cells in human umbilical cord blood express nerve growth factor  [ **NGFB** ]  and its specific receptor TrkA  [ **NTRK1** ] .  Ref: 12620652 J Neuroimmunol, 2003
- We have examined the effect of global transient cerebral ischemia, evoked in rat by 10 min of cardiac arrest, upon the changes in the cellular expression of two nerve growth factor  [ **NGFB** ]  NGF  [ **NGFB** ]  receptors TrkA  [ **NTRK1** ]  and p75 in the hippocampus.  Ref: 12849738 Neuroscience, 2003
- We previously showed that intraventricular administration of a synthetic peptide that interferes with nerve growth factor  [ **NGFB** ]  stability and thus its binding to TrkA  [ **NTRK1** ]  and p75 NTR receptors suppressed kindling and sprouting.  Ref: 12831869 Neuroscience, 2003
- The TrkA  [ **NTRK1** ]  NGF  [ **NGFB** ]  receptor is essential for the survival and differentiation of sensory neurons.  Ref: 12810599 Development< MedlineTA>Development, 2003
- After 2 weeks of treatment of PLA cells with isobutylmethylxanthine, indomethacin, and insulin, about 20 to 25 percent of the cells differentiated into cells with typical neural morphologic characteristics, accompanied by increased expression of NSE, vimentin, and the nerve growth factor  [ **NGFB** ]  receptor trk A  [ **NTRK1** ] .  Ref: 12711954 Plast Reconstr Surg, 2003
- Thus, a chimeric receptor was created that possessed the extracellular and transmembrane domains of the nerve growth factor  [ **NGFB** ]  NGF  [ **NGFB** ]  receptor TrkA  [ **NTRK1** ]  and the cytoplasmic domain of PDGFRbeta TTbeta.  Ref: 12794304 J Mol Neurosci, 2003
- OBJECTIVE To assess the effects of both TrkA  [ **NTRK1** ]  and p75 NTR on nerve growth factor  [ **NGFB** ]  NGF  [ **NGFB** ]  induced differentiation of neuroblastoma cells.  Ref: 12877805 Chin Med J (Engl), 2003
- Nerve growth factor  [ **NGFB** ]  belongs to a small family of proteins whose binding at the Trk  [ **NTRK1** ]  and p75 NTR transmembrane receptors triggers a cascade of signaling events that give rise to neurotrophic responses in neuronal cells and in vivo.  Ref: 12769635 Curr Drug Target CNS Neurol Disord, 2002
- The expression of NGF  [ **NGFB** ]  and TrkA  [ **NTRK1** ] .mRNA was regulated by interleukin 1beta.  Ref: 12593600 J Periodontol, 2003
- In this review we discuss the structure and functions of neurotrophins such as nerve growth factor  [ **NGFB** ] , and the role of these proteins and their tyrosine kinase Trk  [ **NTRK1** ]  receptors in the aetiology and therapy of such diseases.  Ref: 12787319 Neuropathol Appl Neurobiol, 2003
- The present study examined the effects of CHHg on I nerve growth factor  [ **NGFB** ]  binding to the TrkA  [ **NTRK1** ]  receptor, NGF  [ **NGFB** ]  induced activation of the TrkA  [ **NTRK1** ]  receptor, and neurite outgrowth in an in vitro model of differentiation using PC12 cells.  Ref: 12644250 Brain Res Dev Brain Res, 2003
- The effects of NGF  [ **NGFB** ]  on glutamate release and Ca2 i elevation were prevented by an anti TrkA  [ **NTRK1** ]  receptor monoclonal antibody.  Ref: 12696558 Neuropharmacology, 2003
- PURPOSE The purpose is to analyze the possible correlation between expression and activation of the high affinity nerve growth factor  [ **NGFB** ]  NGF  [ **NGFB** ]  receptor TrkA  [ **NTRK1** ] , cell cycle protein expression, and disease outcome in serous ovarian carcinoma.  Ref: 12796393 Clin Cancer Res< MedlineTA>Clin Cancer Res, 2003
- In the present study, the effect of signaling generated from the Erb B2 homodimer as a result of transcription of the MUC1 gene was investigated in human mammary epithelial cell lines MTSV1 7 and Hb2 stably transfected with a pBAT trk  [ **NTRK1** ]  neu construct in which the extracellular domain of Erb B2 was replaced with the corresponding domain from the nerve growth factor  [ **NGFB** ]  NGF  [ **NGFB** ]  receptor.  Ref: 12798775 Cell Biol Int, 2003
- In particular, the putative role of NGF  [ **NGFB** ]  and its receptor TrkA  [ **NTRK1** ]  in immune system homeostasis has become a much studied topic.  Ref: 12892403 J Anat, 2003
- CONCLUSION Down regulated expression of NGF  [ **NGFB** ]  family and their receptors Trk  [ **NTRK1** ]  family mRNA in gastric cancer is confirmed.  Ref: 12854135 World J Gastroenterol, 2003
- Our results showed a marked expression of TrkA  [ **NTRK1** ]  and NGF  [ **NGFB** ]  in cord blood CD34 cells.  Ref: 12620652 J Neuroimmunol, 2003
- The cotransfection of the two genes into this cell line resulted in the more rapid and more apparent morphological changes than single TrkA  [ **NTRK1** ]  transfected cells after NGF  [ **NGFB** ]  treatment.  Ref: 12877805 Chin Med J (Engl), 2003
- In this study, we investigated whether hematopoietic stem cells HSC and progenitors present in human cord blood can express nerve growth factor  [ **NGFB** ]  NGF  [ **NGFB** ]  specific receptors, TrkA  [ **NTRK1** ]  and p75.  Ref: 12620652 J Neuroimmunol, 2003
- Individual and combined effects of TrkA  [ **NTRK1** ]  and p75NTR nerve growth factor  [ **NGFB** ]  NGF  [ **NGFB** ]  receptors A role for the high affinity receptor site.  Ref: 12702729 J Biol Chem< MedlineTA>J Biol Chem, 2003
- Quantitative in situ hybridization was used to detect changes in neuronal PACAP mRNA expression and to correlate this expression with neuronal mRNA expression of the NGF  [ **NGFB** ]  receptor tyrosine kinase trk A  [ **NTRK1** ] .  Ref: 12890505 Neuroscience, 2003
- In this work the expression and anatomical localization of NGF  [ **NGFB** ] , BDNF and NT 3 and of corresponding high affinity receptors TrkA  [ **NTRK1** ] , TrkB full length and truncated TR isoforms, TrkC and of the low affinity p75 receptor were assessed in surgical samples from adult human lung by reverse transcriptase polymerase chain reaction RT PCR, Western blot and immunohistochemistry.  Ref: 12791675 Am J Respir Cell Mol Biol< MedlineTA>Am J Respir Cell Mol Biol, 2003
- Both of NGF  [ **NGFB** ]  family and their receptors Trk  [ **NTRK1** ]  family mRNA were expressed in gastric cancer and normal gastric mucosa.  Ref: 12854135 World J Gastroenterol, 2003
- Taken together, our results indicate that retrogradely transported NGF  [ **NGFB** ]  TrkA  [ **NTRK1** ]  complexes promote sympathetic neuron survival.  Ref: 12848932 Neuron, 2003
- Molecular Dynamics Simulations of the NGF  [ **NGFB** ]  TrkA  [ **NTRK1** ]  Domain 5 Complex and Comparison with Biological Data.  Ref: 12668437 Biophys J, 2003
- In order to test the functional activity of trkA  [ **NTRK1** ]  receptors in the sensory ganglia of adult animals in vivo, we examined the ability of a nerve growth factor  [ **NGFB** ]  expressing recombinant replication defective herpes simplex virus based vector to prevent the selective degeneration of large sensory fibres caused by intoxication with pyridoxine.  Ref: 12603263 Eur J Neurosci, 2003
- On the contrary, in nondiferentiated TrkA  [ **NTRK1** ]  NGF  [ **NGFB** ]  brought about a 2.5 fold increase of choline acetyltransferase.  Ref: 12675131 Neurochem Res, 2003
- Retrovirus mediated transfer of the beta NGF  [ **NGFB** ]  gene into NET cells activated TrkA  [ **NTRK1** ]  and consistently decreased their proliferative responses to insulin like growth factor IGF I, IGF II, fibroblast growth factor 2, and epidermal growth factor EGF, down regulating EGF and IGF I binding sites.  Ref: 12671999 J Neurosci Res, 2003
- In addition, a new interaction in the TrkA  [ **NTRK1** ]  p75NTR heteromeric receptor signal transduction network was revealed, namely that NGF  [ **NGFB** ]  induced activation of the MAPK pathway appears to inhibit the parallel NGF  [ **NGFB** ]  induced Akt pathway.  Ref: 12702729 J Biol Chem< MedlineTA>J Biol Chem, 2003
- Western blot analysis confirmed these results, showing that the lacrimal gland expresses NGF  [ **NGFB** ] , BDNF, NT3, and NT4 as well as the NTrs TrkA  [ **NTRK1** ] , TrkB, and TrkC and the p75 protein.  Ref: 12882781 Invest Ophthalmol Vis Sci, 2003
- In TrkA  [ **NTRK1** ]  cells, NGF  [ **NGFB** ]  100 ng ml affected neither choline acetyltransferase nor morphology but depressed pyruvate dehydrogenase activity by about 30%.  Ref: 12675131 Neurochem Res, 2003
- In contrast, disruption of TrkA  [ **NTRK1** ]  activity exclusively in proximal axonal segments affects neither retrograde NGF  [ **NGFB** ]  TrkA  [ **NTRK1** ]  signaling in cell bodies nor neuronal survival.  Ref: 12848932 Neuron, 2003
- RT PCR showed positive bands at the appropriate sizes for NGF  [ **NGFB** ] , BDNF, NT3, and NT4, and for the receptors TrkA  [ **NTRK1** ] , TrkB, TrkC, and p75.  Ref: 12882781 Invest Ophthalmol Vis Sci, 2003
- None of these correlated with NGF  [ **NGFB** ]  or TrkA  [ **NTRK1** ]  expression or activity.  Ref: 12796393 Clin Cancer Res< MedlineTA>Clin Cancer Res, 2003
- Human periodontal ligament cells, human gingival fibroblasts, and human gingival keratinocytes expressed mRNA for NGF  [ **NGFB** ]  and TrkA  [ **NTRK1** ] .  Ref: 12593600 J Periodontol, 2003
- Here we study energetics and dynamics of the NGF  [ **NGFB** ]  TrkA  [ **NTRK1** ]  complex by carrying out multinanosecond molecular dynamics simulations, accompanied by electrostatic calculations based on the Poisson Boltzmann equation.  Ref: 12668437 Biophys J, 2003
- Binding of neurotrophins to p75 induced internalization at a rate approximately three times slower than that of transferrin and NGF  [ **NGFB** ]  TrkA  [ **NTRK1** ]  complexes in the same cells.  Ref: 12716928 J Neurosci, 2003
- Consistent with the presence of trkB but not trkA  [ **NTRK1** ] , BDNF causes rapid phosphorylation of MAP kinases ERK1 and ERK2, but NGF  [ **NGFB** ]  does not.  Ref: 12672017 J Neurobiol, 2003
- In a flow cytometry study, the nerve growth factor  [ **NGFB** ]  mediated antiapoptotic effect was facilitated by AMPA, with an induction of TrkA  [ **NTRK1** ] , but not p75 NTR expression.  Ref: 12595756 J Biomed Sci< MedlineTA>J Biomed Sci, 2003
